# Causal association between plasma metabolites and diverse autoimmune diseases: a two-sample bidirectional mendelian randomization study

**DOI:** 10.3389/fimmu.2024.1437688

**Published:** 2024-11-07

**Authors:** Xiwen Yuan, Peiyan Yang, Jiapeng Hu, Dixin Cai, Baoshan Hu, Gang Rui, Zhiming Lin

**Affiliations:** ^1^ Department of Orthopedics, The First Affiliated Hospital of Xiamen University, School of Medicine, Xiamen University, Xiamen, China; ^2^ State Key Laboratory of Cellular Stress Biology, School of Medicine, Faculty of Medicine and Life Sciences, Xiamen University, Xiamen, China; ^3^ The Third Clinical Medical College, Fujian Medical University, Fuzhou, China; ^4^ Department of Pediatrics, Shengjing Hospital of China Medical University, Shenyang, China

**Keywords:** Mendelian randomization, plasma metabolites, autoimmune diseases, inflammatory bowel disease, multiple sclerosis, type 1 diabetes, systemic lupus erythematosus, rheumatoid arthritis

## Abstract

**Background:**

Autoimmune diseases (ADs) are a category of conditions characterized by misrecognition of autologous tissues and organs by the immune system, leading to severe impairment of patients’ health and quality of life. Increasing evidence suggests a connection between fluctuations in plasma metabolites and ADs. However, the existence of a causal relationship behind these associations remains uncertain.

**Methods:**

Applying the two-sample mendelian randomization (MR) method, the reciprocal causality between plasma metabolites and ADs was analyzed. We took the intersection of two metabolite genome-wide association study (GWAS) datasets for GWAS-meta and obtained 1,009 metabolites’ GWAS data using METAL software. We accessed GWAS summary statistics for 5 common ADs, inflammatory bowel disease (IBD), multiple sclerosis (MS), type 1 diabetes (T1D), systemic lupus erythematosus (SLE) and rheumatoid arthritis (RA) from published GWAS data. MR analyses were performed in discovery and replication stage simultaneously. Meanwhile, the reverse MR analysis was conducted to investigate the possibility of reverse causal association. Furthermore, a series of sensitivity analyses were conducted to validate the robustness of the results. These statistical analyses were conducted using R software. Finally, the web version of MetaboAnalyst 5.0. was applied to analyze metabolic pathways. Ultimately, we conducted ELISA assays on plasma samples from patients to validate the results.

**Results:**

4 metabolites were identified to have causal relationships with IBD, 2 metabolites with MS, 13 metabolites with RA, and 4 metabolites with T1D. In the reverse MR analysis, we recognized causality between SLE and 22 metabolites, IBD and 4 metabolites, RA and 22 metabolites, and T1D and 37 metabolites. Additionally, 4 significant metabolic pathways were identified in RA by metabolic pathway analysis in the forward MR analysis. Correspondingly, in the reverse, 11 significant metabolic pathways in RA, 8 in SLE, and 4 in T1D were obtained using identical approaches. Furthermore, the protective role of glutamate was confirmed through ELISA assays.

**Conclusions:**

Our research established a reciprocal causality between plasma metabolites and ADs. Furthermore, diverse metabolic pathways correlated with ADs were uncovered. Novel insights into the prediction and diagnosis were provided, as well as new targets for precise treatment of these conditions were discovered.

## 1 Introduction

Autoimmune diseases (ADs) are a group of disorders in which the immune system mistakenly targets and destroys healthy tissues and organs within the body. This can result in inflammation, tissue damage, and dysfunction of multiple organ systems (1). The number of people affected by autoimmune diseases is approximately 10% of the population worldwide and is expected to continue to increase globally (2). Autoimmune diseases frequently impact multiple organs within the body, posing significant challenges and financial burdens in terms of treatment. This is undoubtedly a heavy pressure on both individuals and society (3). The etiology of ADs is believed to involve an intricate combination of hereditary, external, hormonal, mental stress and immune factors (4). However, the exact causes of autoimmune diseases are currently not understood completely.

Plasma encompasses a spectrum of small molecule metabolites that frequently engage in diverse biological processes and contribute to the pathogenesis of various diseases. The application of these metabolites in the diagnosis of human diseases is increasingly recognized and is a focal point of contemporary medical research (5, 6). Plasma metabolites play a significant role in their native state. Valine, as an example, is an essential amino acid in its natural state, playing a crucial role in protein synthesis (7). Homocitrulline, as another example, is a derivative of an amino acid that facilitates in the cellular removal of excess ammonia, thereby maintaining ammonia homeostasis (8). Although the pathogenesis of ADs is still unclear, in recent years, more and more evidence has shown that metabolic abnormalities are closely related to the development of ADs. For example, a metabolomics study on systemic lupus erythematosus (SLE) found a significant decrease in many serum metabolites including valine (9). Similarly, in another study on amino acid analysis in patients with inflammatory bowel disease (IBD), significant changes in valine levels were also observed (10). At the same time, previous studies have demonstrated significant variations of plasma homocitrulline level in Type 1 diabetes (T1D) patients with various symptoms (11). These previous studies have suggested that there is some correlation between metabolites and ADs, and even that metabolite levels may be related to the severity of disease symptoms. Nevertheless, due to the presence of numerous confounding factors, it is hard to determine whether the potential causal relationship exist between plasma metabolites and ADs through traditional cross-sectional studies.

With the development of high-throughput technology, it has become feasible to simultaneously evaluate a vast number of plasma metabolites. Metabolomics is progressively assuming a crucial role in the investigation of disease occurrence and progression in ADs (12). Genome-wide association study (GWAS) greatly facilitates the comprehensive investigation of the underlying genetic factors contributing to complex diseases. Mendelian randomization (MR), a statistical method, provides confounder-free estimates effectively. It employs genetic variation as an unbiased instrumental variable (IV) to study the casual relationship between exposure and outcome (13). MR has been proved to be a powerful statistical approach, as the outcomes are less susceptible to be affected by unknown confounding variables or reverse causality (14). MR analysis has been extensively utilized in diverse situations, encompassing some studies related to plasma metabolites or ADs (15, 16).

In this study, we conducted a comprehensive two-sample bidirectional MR analysis to (1) assess the causality of human plasma metabolites on 5 ADs, including IBD, multiple sclerosis (MS), T1D, SLE and rheumatoid arthritis (RA). (2) reversely determine the causal relationship of 5 ADs on plasma metabolites. (3) explore potential metabolic pathways that may contribute to elucidate the mechanisms of ADs.

## 2 Materials and methods

### 2.1 Study design

**Figure 1:**
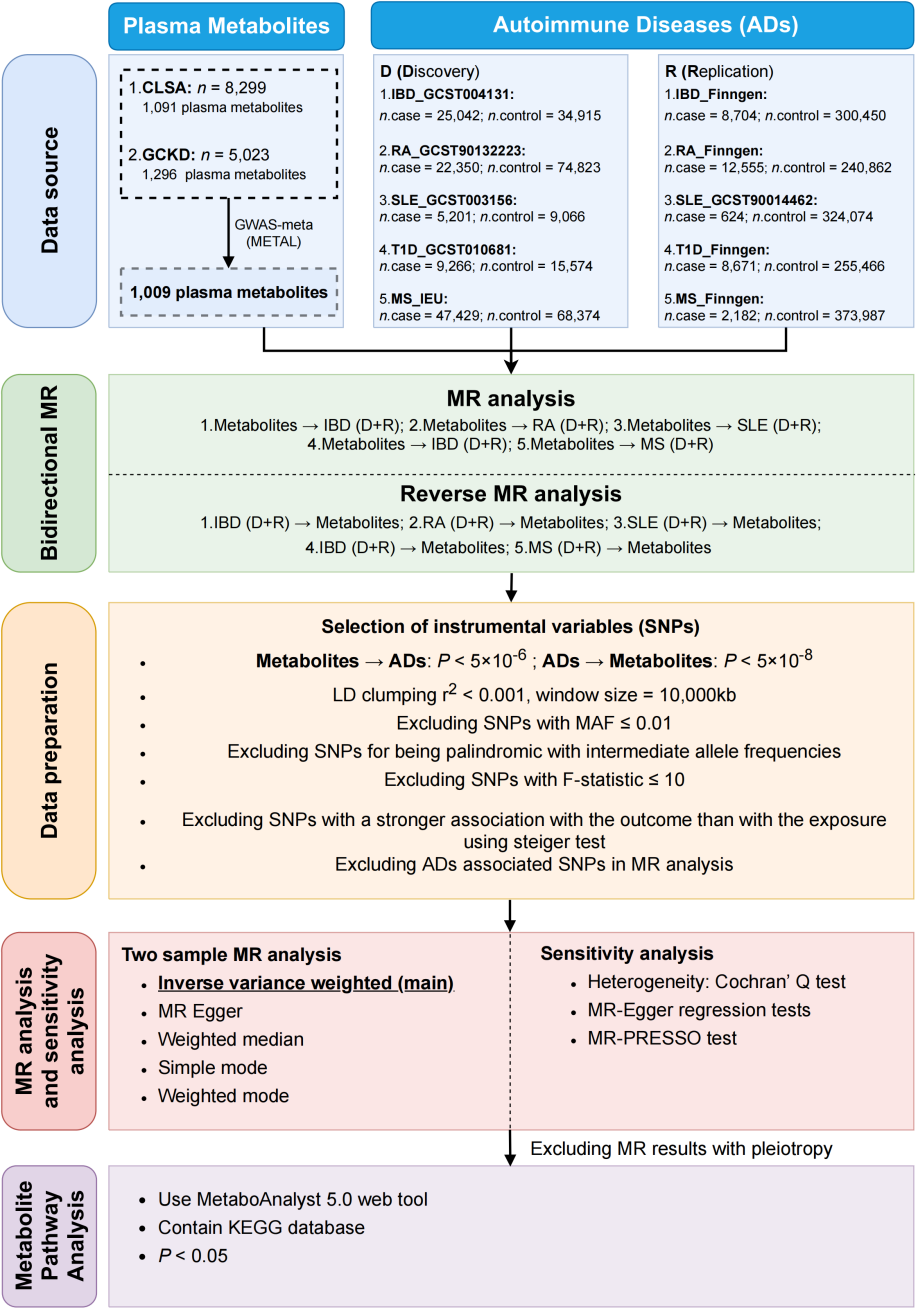
The summary of the research process.

We conducted a comprehensive evaluation of the potential causal relationship between human plasma metabolites and the likelihood of ADs using a bidirectional two-sample MR design. A well-executed MR study should adhere to three fundamental assumptions: (1) instrumental variable assumption—the genetic variation should exhibit a direct association with exposure; (2) independence assumption—the genetic variant is randomly assigned and is independent of any confounding factors that may affect the outcome; (3) exclusion restriction assumption—the genetic variation have no direct effect on the outcome, and affect the outcome variable only through the effect of the exposure variable. To ensure data integrity, we acquired individual GWAS datasets for plasma metabolites and ADs, thereby avoiding overlap of samples. The summary of this study was depicted in **Figure 1**. The overview of the research workflow.

### 2.2 Human plasma metabolites GWAS summary statistics

The genetic statistics for plasma metabolites come from two different GWAS data: One is from a German Chronic Kidney Disease (referred to as GCKD below) involving a cohort of 5023 German individuals, and summary statistics for study GCST90264176-GCST90266872 (17) were obtained from the NHGRI-EBI GWAS Catalog (18) on 05/06/2023. We only selected plasma metabolites for further study and urine metabolites were excluded. The other is from a Canadian Longitudinal Study of Aging (referred to as CLSA below) including 8,299 Canadian individuals, and summary statistics for study GCST90199621-GCST90200711 (19) were also acquired from the NHGRI-EBI GWAS Catalog (18) on 05/06/2023. These are the two large-scale GWAS data published to date for plasma metabolites which were published in 2023 and they shared the same platform called Metabolon HD4 thus perfectly increasing the data consistency. We meta-analyzed summary statistics from GCKD and CLSA cohorts using METAL software (20).

### 2.3 Autoimmune diseases GWAS summary statistics (discovery samples)

During the discovery phase, summary statistics for each of the 5 ADs were obtained from the NHGRI-EBI GWAS Catalog: GCST004131 (25,042 IBD cases and 34,915 controls) (21), GCST90132223 (22,350 RA cases and 74,823 controls) (22), GCST003156 (5,201 SLE cases and 9,066 controls) (23) and GCST010681 (9,266 T1D cases and 15,574 controls) (24). In order to facilitate correspondence with diseases, these GWAS datasets were named as followed: IBD_GCST004131, RA_GCST90132223, SLE_GCST003156 and T1D_GCST010681. Data for MS was obtained from the IEU Open GWAS Project, so we called MS_IEU (47,429 MS cases and 68,374 controls) below (25).

### 2.4 Autoimmune diseases GWAS summary statistics (replication samples)

For the replication phase, the FinnGen study provided the replication outcome samples for IBD, RA, T1D, and MS. To distinguish these GWAS datasets, we have all prefixed them with the name of the disease: IBD_Finngen (8,704 IBD cases and 300,450 controls), RA_Finngen (12,555 RA cases and 240,862 controls), T1D_Finngen (8,671 T1D cases and 255,466 controls) and MS_Finngen (2,182 MS cases and 373,987 controls) (26). The samples for SLE were obtained from the NHGRI-EBI GWAS Catalog: GCST90014462 (624 SLE cases and 324,074 controls), then we called it SLE _GCST90014462 below (27).

### 2.5 Selection of IVs

Initially, we identified single nucleotide polymorphisms (SNPs) for each plasma metabolite, which met the association threshold of *P* < 5 × 10^–6^ (28–30). The commonly employed threshold for genome-wide significance, *P* < 5 × 10^–8^ is applied to select genetic instruments. However, imposing such conditions will result in a significant reduction in the quantity of metabolites that can be analyzed. To obtain a more comprehensive evaluation, we relaxed the threshold to *P* < 5 × 10^–6^. In reverse, as a standard practice, SNPs were selected with association thresholds at *P* < 5 × 10^–8^ for each AD analyzed. Next, link-age disequilibrium between the SNPs was calculated, and we only retained those SNPs with a r^2^ < 0.001 (clumping window size = 10,000 kb). In addition, SNPs with minor allele frequency (MAF) ≤ 0.01, SNPs with alleles that form a palindrome and have intermediate allele frequencies and SNPs with F-statistics ≤ 10 were all excluded. Then, we removed SNPs associated with disease and potential confounding factors, which were checked in the Phenoscanner of R software (31, 32). Finally, SNPs that exhibited a stronger correlation with the outcome rather than with the exposure were removed based on the Steiger Test (33).

### 2.6 MR analyses

In our MR analyses, the standard inverse variance weighted (IVW) method, Simple mode test, MR Egger test, Weighted mode test and Weighted-median method were employed to evaluate the causal relationship between plasma metabolites and ADs, and IVW method is the primary and most significant approach among them. Subsequently, we carried out diverse sensitivity analyses simultaneously to ensure the reliability and robustness of the results: (1) Cochran’s Q was calculated to assess heterogeneity among the individual causal correlation, where pleiotropy is considered present when *P* < 0.05. (2) MR-Egger and MR-PRESSO regression tests were applied to examine the potential impact of horizontal pleiotropy. In both methods, we delete the data with a *P* < 0.05.

Statistical analyses were conducted using R software (version 4.3.1). Forest plot was performed using package “forestploter” (version 1.1.0) (https://CRAN.R-project.org/package=forestploter). We conducted MR analyses using the “TwoSampleMR” package (version 0.5.6) (34). We used “ieugwasr” package (version 0.1.5) (https://github.com/MRCIEU/ieugwasr) to analyze linkage disequilibrium.

### 2.7 Metabolic pathway analyses

Metabolic pathways were investigated using the web version of MetaboAnalyst 5.0. (https://www.metaboanalyst.ca/) (35). To identify potential metabolite groups or pathways that may be associated with ADs’ biological process, pathway analyses modules and functional enrichment analyses were employed. In this study, the Kyoto Encyclopedia of Genes and Genomes (KEGG) database was utilized, and a significance level of 0.05 was applied for pathway analyses.

### 2.8 Patients

This study has been approved by the Ethics Committee of The First Affiliated Hospital of Xiamen University (Xiamen, China; approval number: XMYY-2022KY121). Informed consent was obtained from all patients. Plasma samples were collected from a control group of 12 patients without ADs who had internal fixation removed (6 males, 6 females), as well as two groups of patients who underwent joint replacement surgery: one group with 12 patients with RA (4 males, 8 females), and another group with 12 patients with SLE (3 males, 9 females).

### 2.9 Glutamate ELISA

Glutamate concentrations were quantified in human plasma according to manufacturer’s instructions (Glutamate ELISA kit KA1909, Novus Biologicals).

### 2.10 Statistical analysis

Experimental data analysis was conducted using SPSS v29.0 (SPSS Inc.) or GraphPad Prism v10.0.2 (GraphPad Inc.). Data were tested for normality and equal variances, followed by 2-sample, 2-tailed t tests or 3-way ANOVA with Dunnett’s *post hoc* tests. *P* < 0.05 was considered significant. Mean ± SD or box plots representing interquartile range, median, and all data points are presented in the figures.

## 3 Results

### 3.1 Selection of IVs

Our workflow is summarized in **Figure 1**. The genetic statistics for plasma metabolites come from two different GWAS data, each with 1,296 and 1,091 metabolites, respectively. 1,009 metabolites were obtained after intersection using METAL software. To investigate the impact of metabolites on diseases, we selected SNPs as IVs after a set of quality assurance procedures. To be specific, in the discovery stage, for IBD, 683 SNPs (*P* < 5.0 × 10^−6^, r^2^ < 0.001) involved with 64 metabolites were extracted from IBD_GCST004131. In the case of RA, 1,489 SNPs associated with 97 metabolites were selected from RA_GCST90132223. For SLE, 540 SNPS were selected from SLE_GCST003156, covering 55 metabolites. Regarding T1D, a total of 933 SNPs were chosen from T1D_GCST010681, encompassing 69 metabolites. Lastly, in the case of MS, 357 SNPs related to 47 metabolites were chosen from the MS_IEU study. Then, in the replication stage, the same screening criteria for selecting IVs are applied (**Supplementary Tables S1–S10**).

At the same time, we also wanted to explore the effects of autoimmune diseases on plasma metabolites, so a reverse MR analysis was performed. Among the SNPs associated with each genus, those that reached the locus-wide significance threshold (*P* < 5.0 × 10^−8^) were selected as potential IVs (**Supplementary Tables S11–S20**). Statistically, all F-statistics for the validity test were all above the standard threshold of 10, indicating a strong genetic instrument.

### 3.2 Causal effects of plasma metabolites on autoimmune diseases

**Figure 2:**
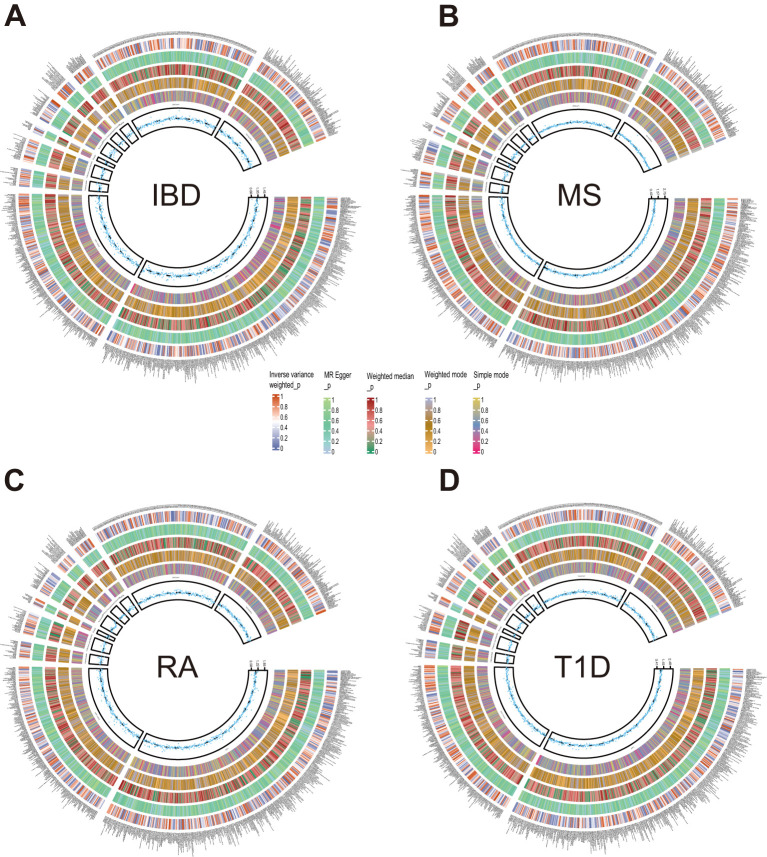
Circle diagrams of the discovery sample in the forward MR analysis. The complete results of the forward MR analysis, showing the causal effects of plasma metabolites on ADs. **(A)** IBD, **(B)** MS, **(C)** RA, **(D)** T1D. Five statistical methods, respectively, IVW, MR Egger, Weighted median, Weighted mode, and Simple mode, are represented by five circles from inner to outer.

Next, we employed five methodologies, respectively, IVW, MR Egger, Weighted median, Weighted mode, and Simple mode, to assess the causal associations of plasma metabolites on ADs as shown in **Figure 2** and **Supplementary Figure S1**. During the discovery phase, 23 diverse metabolites were preliminarily identified by IVW method to have significant causal relationship with these autoimmune diseases, including 3 unknown components and the remaining 20 known metabolites involved in 4 metabolic pathways. Specifically, they were as follows:

4 metabolites from 2 pathways for IBD: 2-hydroxy-4-(methylthio)butanoic acid (odds ratio (OR) = 1.095; 95% confidence interval (95% CI) 1.025-1.171; *P* = 0.008), sphingomyelin (d18:2/16:0, d18:1/16:1)* (OR = 0.704; 95% CI 0.557-0.890; *P* = 0.003), sphingosine (OR = 0.886; 95% CI 0.801-0.979; *P* = 0.018), X-12839 (OR = 1.161; 95% CI 1.049-1.285; *P* = 0.004).

2 metabolites from 2 pathways for MS: N-acetylcitrulline (OR = 1.526; 95% CI 1.183-1.968; *P* = 0.001), threonate (OR = 0.757; 95% CI 0.589-0.972; *P* = 0.029).

13 metabolites from 2 pathways for RA: 1-(1-enyl-palmitoyl)-2-arachidonoyl-GPC (P-16:0/20:4)* (OR = 1.160; 95% CI 1.066-1.262; *P* = 0.001), 1-arachidonoyl-GPC (20:4n6)* (OR = 1.184; 95% CI 1.050-1.335; *P* = 0.006), 1-oleoyl-2-linoleoyl-GPE (18:1/18:2)* (OR = 0.900; 95% CI 0.845-0.958; *P* = 0.001), 1-palmitoleoyl-2-linolenoyl-GPC (16:1/18:3)* (OR = 0.885; 95% CI 0.797-0.982; *P* = 0.021), 1-palmitoyl-2-linoleoyl-GPC (16:0/18:2) (OR = 0.615; 95% CI 0.423-0.895, *P* = 0.011), 1-palmitoyl-2-linoleoyl-GPE (16:0/18:2) (OR = 0.898; 95% CI 0.838-0.961; *P* = 0.002), 1-stearoyl-2-arachidonoyl-GPC (18:0/20:4) (OR = 1.317; 95% CI 1.172-1.480; *P* = 0.000), 1-stearoyl-2-linoleoyl-GPE (18:0/18:2)* (OR = 0.897; 95% CI 0.816-0.986; *P* = 0.024), 1-stearoyl-2-oleoyl-GPE (18:0/18:1) (OR = 0.893; 95% CI 0.798-1.000; *P* = 0.050), 5alpha-androstan-3beta,17beta-diol monosulfate (2) (OR = 0.890; 95% CI 0.813-0.974; *P* = 0.011), androsterone sulfate (OR = 0.937; 95% CI 0.891-0.985; *P* = 0.011), metabolonic lactone sulfate (OR = 0.946; 95% CI 0.905-0.989; *P* = 0.014), X-26109 (OR = 0.923; 95% CI 0.881-0.967; *P* = 0.001).

4 metabolites from 2 pathways for T1D: beta-hydroxyisovaleroylcarnitine (OR = 0.709; 95% CI 0.531-0.946; *P* = 0.020), 1-palmitoyl-2-dihomo-linolenoyl-GPC (16:0/20:3n3 or 6)* (OR = 0.622; 95% CI 0.427-0.905; *P* = 0.013), glycohyocholate (OR = 1.236; 95% CI 1.045-1.463; *P* = 0.014), X-21310 (OR = 0.653; 95% CI 0.516-0.825; *P* = 0.000).

**Figure 3:**
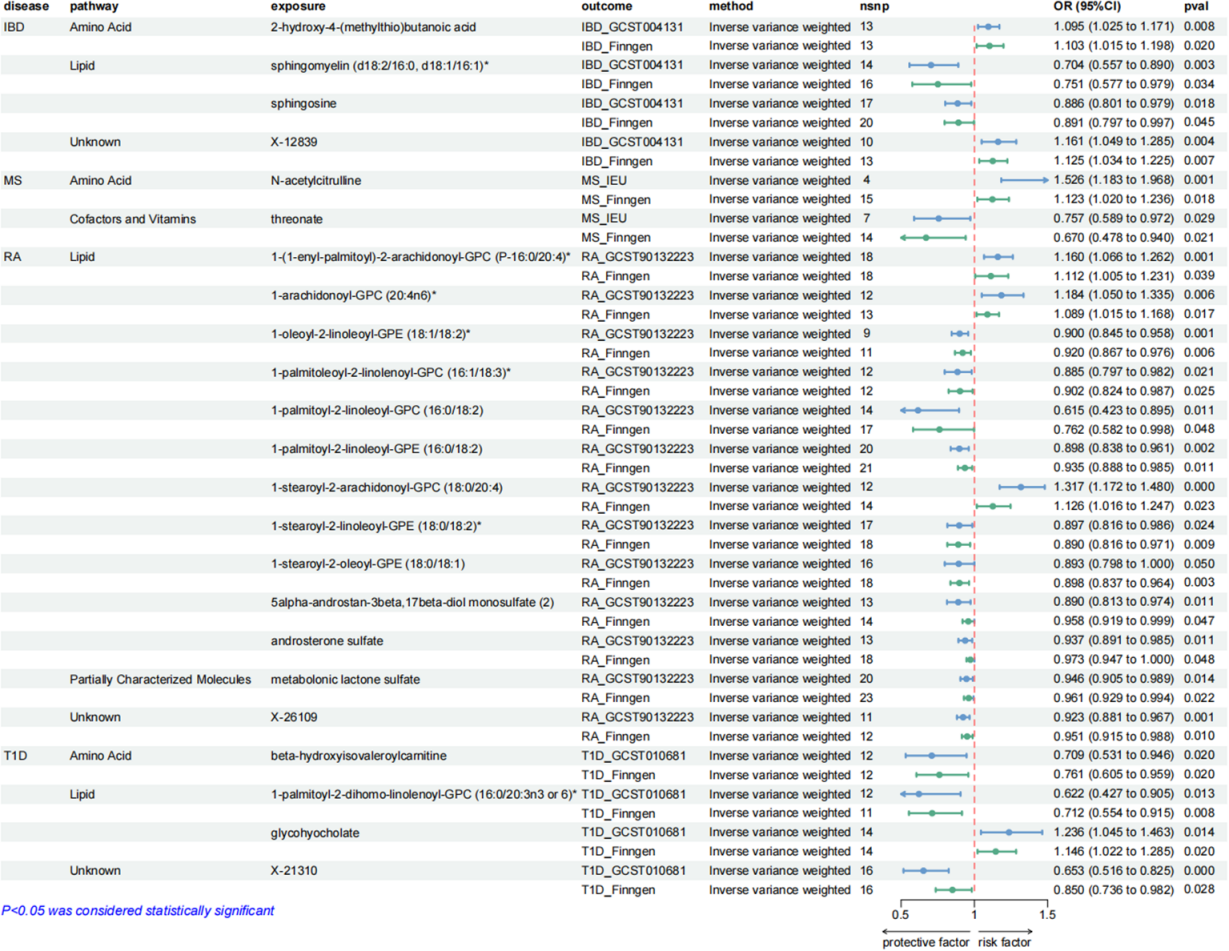
Forest plots showing the causal relationships between plasma metabolites and ADs outcomes. All causal relationships were derived from the fixed-effect IVW analysis and can be supported by both discovery and replication samples.

These causal relationships were further supported by the replication samples, as depicted in **Figure 3**. Two samples were used to enhance the confidence of causal association. We only established a causal relationship between a metabolite and an AD when the metabolite was identified as a protective factor or a risk factor simultaneously by discovery and replication sets, and both sets of results have statistical significance (*P* < 0.05). The graphs showing the intersection of discovery and replication samples in the forward analysis are listed in **Supplementary Figure S3**.

### 3.3 Causal effects of autoimmune diseases on plasma metabolites

**Figure 4:**
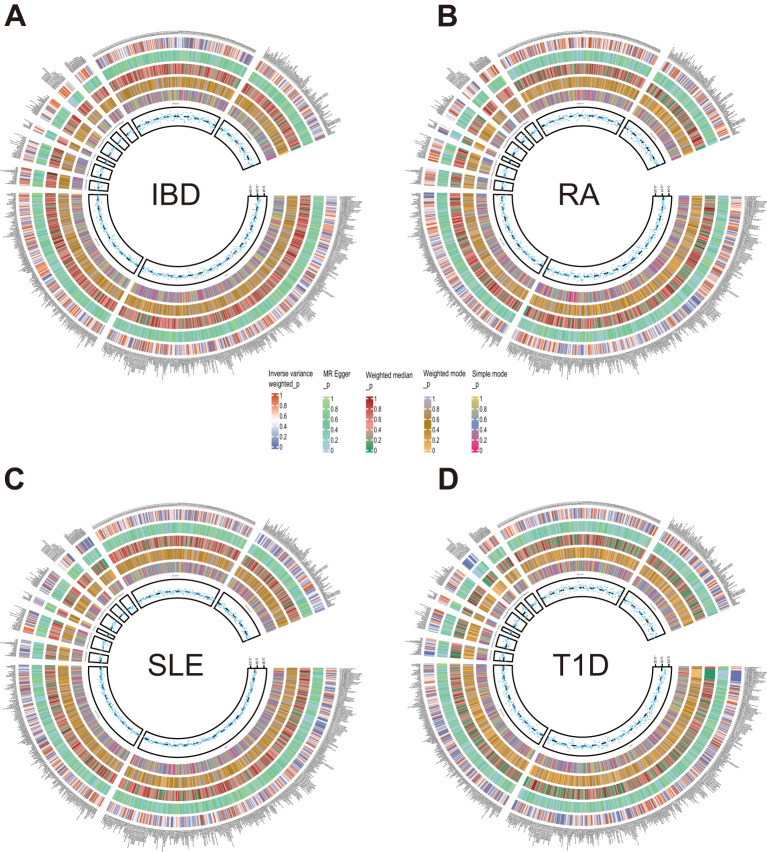
Circle diagrams of the discovery sample in the reverse MR analysis. The complete results of the reverse MR analysis, showing the causal effects of ADs on plasma metabolites. **(A)** IBD, **(B)** RA, **(C)** SLE, **(D)** T1D. Five statistical methods, respectively, IVW, MR Egger, Weighted median, Weighted mode, and Simple mode, are represented by five circles from inner to outer.

After exploring the causal effects of autoimmune diseases on plasma metabolites, we further conducted a reverse MR analysis, as depicted in **Figure 4** and **Supplementary Figure S2**. According to the preliminary identification by IVW, IBD had a causal relationship with 4 metabolites, 2 of which were from the amino acid metabolic pathway and 1 was from the lipid metabolic pathway.

Furthermore, our analysis also demonstrated a significant causal correlation between RA and 22 metabolites. These metabolites encompassed 8 from the amino acid pathways, 8 from the lipid metabolism pathways, 1 from the energy pathways, and 1 from the xenobiotic pathways.

Similarly, SLE was found to have a causal link with 22 metabolites. These metabolites compromised 7 from the lipid metabolism pathways, 4 metabolites from the amino acid pathways, 3 from the nucleotide pathways, 3 from the xenobiotic pathways, 2 from the carbohydrate pathways and 1 from the peptide pathways.

**Figure 5:**
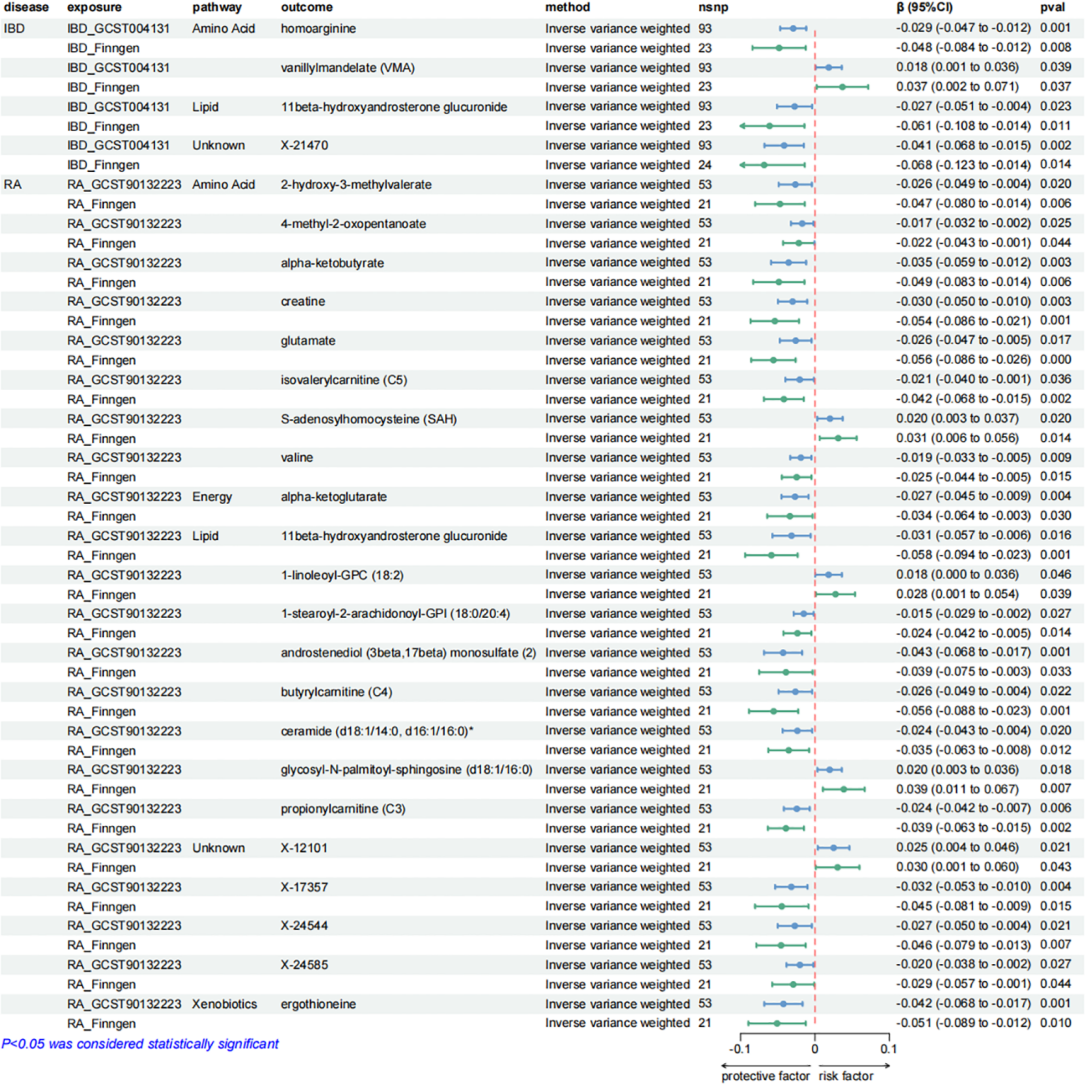
Forest plots presenting the results of reverse MR analysis (1). Causal effects of IBD and RA on plasma metabolites using IVW method, which were supported by both discovery and replication samples.

**Figure 6:**
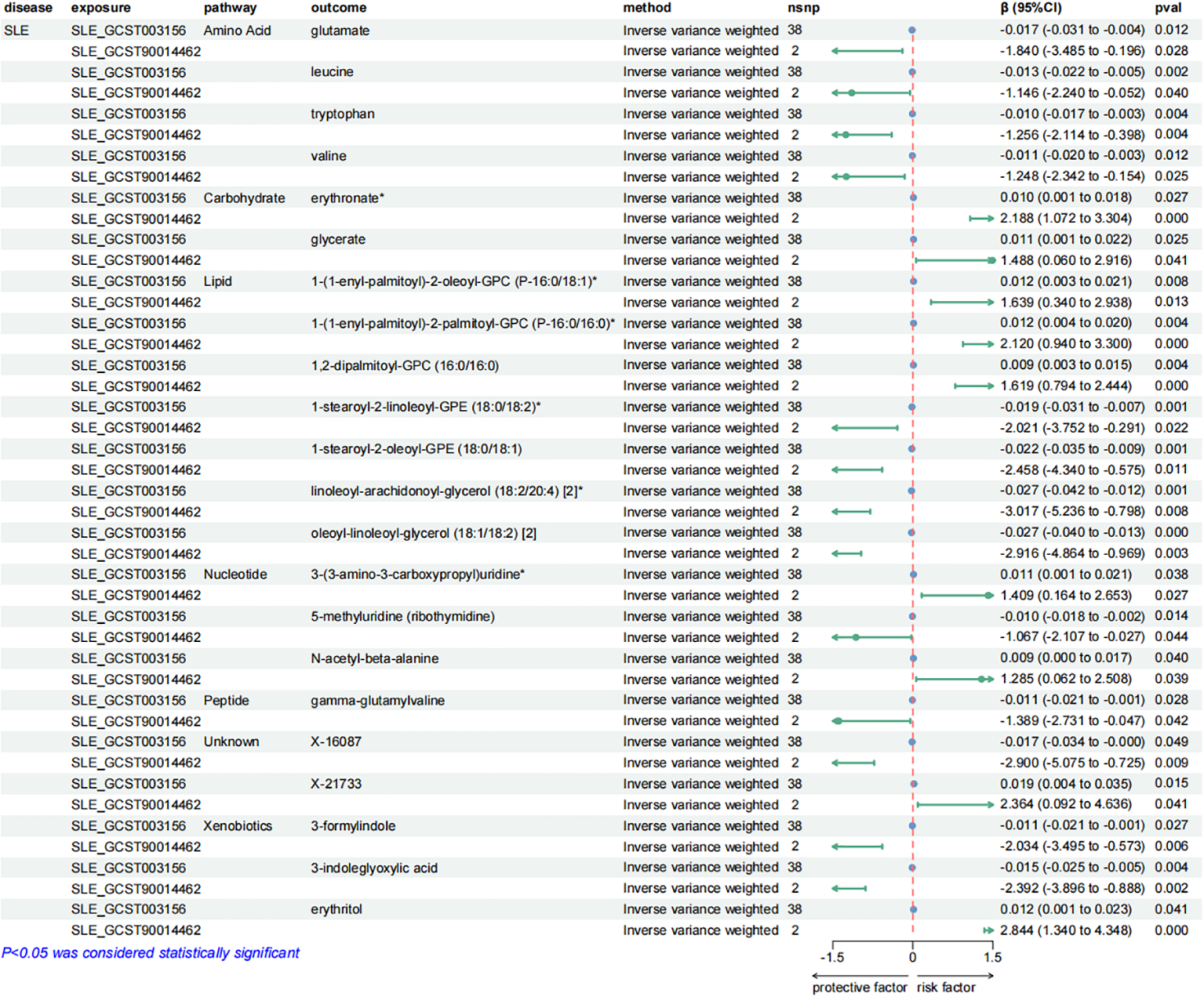
Forest plots presenting the results of reverse MR analysis (2). Causal effects of SLE on plasma metabolites using IVW method, which were supported by both discovery and replication samples.

**Figure 7:**
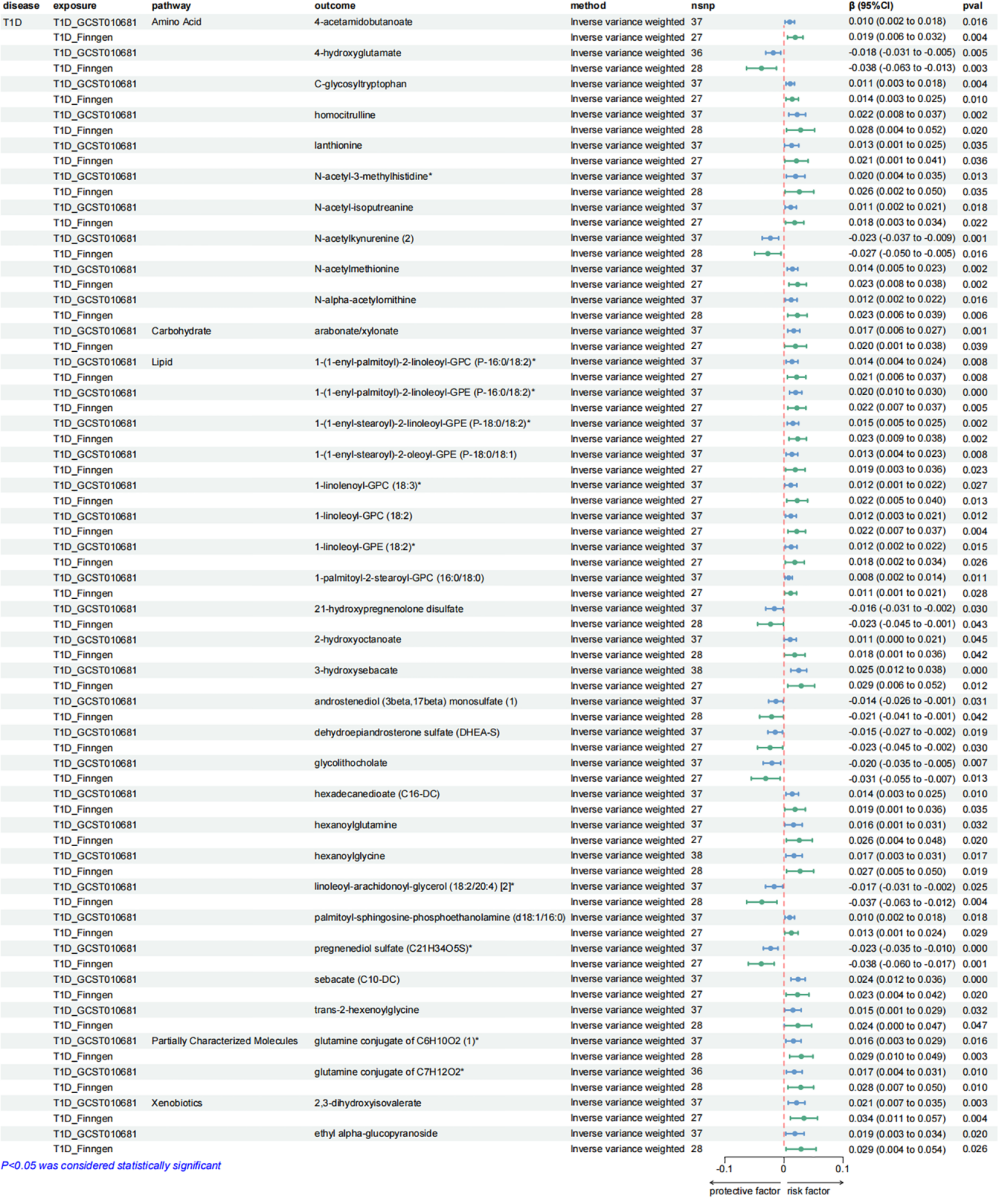
Forest plots presenting the results of reverse MR analysis (3). Causal effects of T1D on plasma metabolites using IVW method, which were supported by both discovery and replication samples.

Additionally, a causal relationship between 37 plasma metabolites and T1D was identified. Among them, 22 were from the lipid metabolism pathways, 10 were from the amino acid pathways, 2 were from the partially characterized molecules pathways, 2 were from the xenobiotic pathways and 1 was from the carbohydrate pathways. **Figures 5**–**7**.

Consistent with the preceding, as was the case with forward MR analysis, these findings were supported by replication samples. The graphs displaying the intersection of discovery and replication samples in the reverse analysis are listed in **Supplementary Figure S4**.

### 3.4 Sensitivity analyses

A series of sensitivity analyses were conducted to minimize the impact of horizontal pleiotropy on the MR estimate as possible, aiming to obtain more robust results. We utilized MR-Egger and MR-PRESSO regression tests to examine the potential horizontal pleiotropy impact. Through screening, samples with *P* < 0.05 in both methods were deleted to provide more reliable results. Horizontal pleiotropy was not found in all relevant metabolites applied in **Figures 3**, **5**–**7**.

At the same time, in order to quantify the heterogeneity among the individual causation effects Cochran’s Q was calculated. Through this test, in the forward MR analysis, Cochran’ Q-derived *P* values indicated the absence of detected heterogeneity in the causal relationships 11 metabolites with RA, 3 metabolites with T1D and all metabolites with IBD and MS (**Supplementary Table S21-S28**). At the same time, in the reverse MR analysis, no heterogeneity was detected in the causal association in 21 metabolites with SLE, 36 metabolites with T1D and all metabolites with IBD and RA (**Supplementary Tables S29–36**).

### 3.5 Metabolic pathway analyses

**Figure 8:**
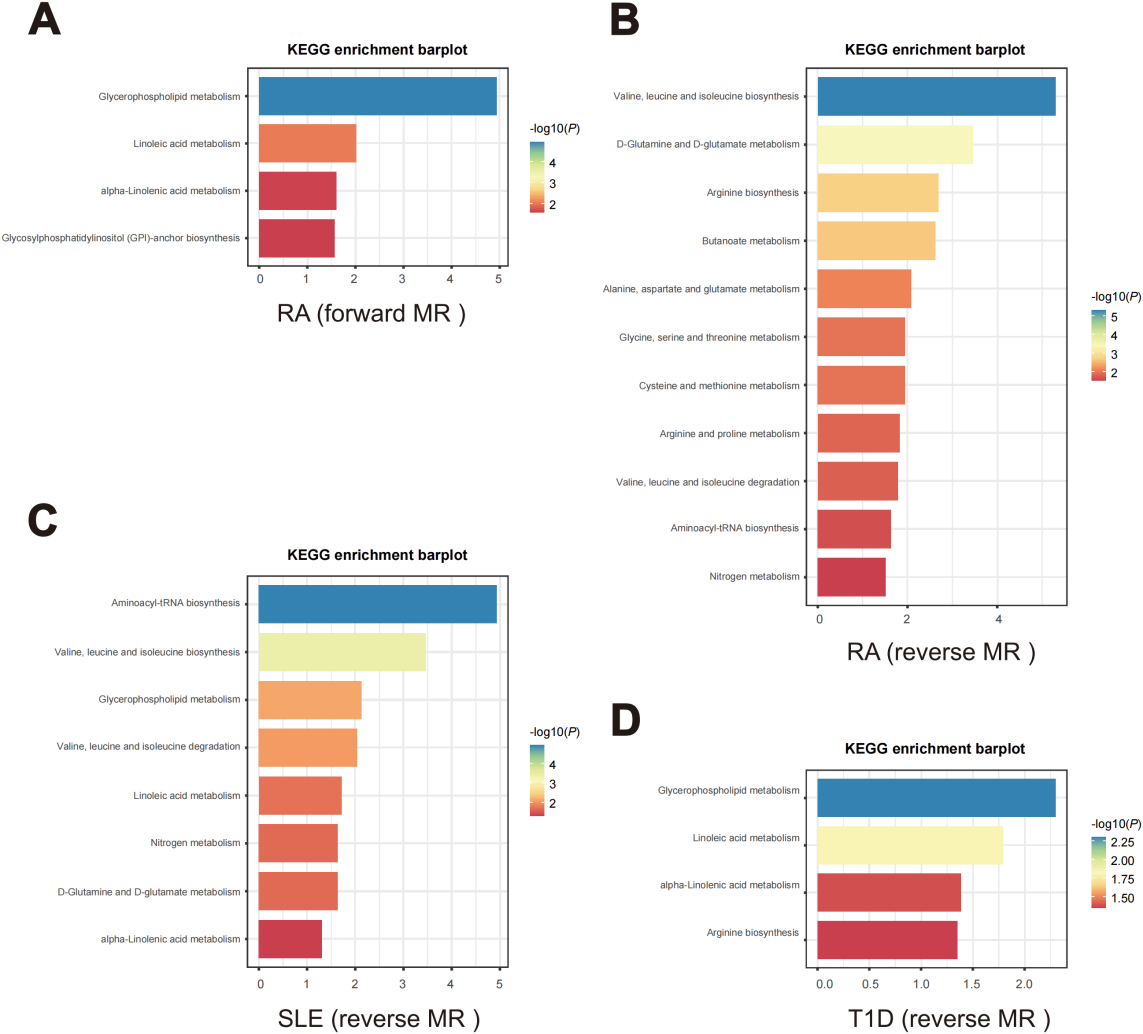
Enriched key metabolic pathways associated with ADs. **(A)** Metabolic pathways in RA identified through the forward MR analysis. **(B-D)** Metabolic pathways in the reverse MR analysis for different ADs. **(B)** RA, **(C)** SLE, **(D)** T1D.

In the forward MR analysis, metabolic pathway analysis revealed that, only in RA, 4 significant metabolic pathways were found, as shown in **Figure 8A**. Our results show that the “Glycosylphosphatidylinositol (GPI)-anchor biosynthesis”, “alpha-Linolenic acid metabolism”, “Linoleic acid metabolism”, and “Glycerophospholipid metabolism” pathways were found to be involved in the pathogenetic process of RA (**Supplementary Table S37**).

In the reverse MR analysis, as shown in **Figures 8B–D**, 14 significant metabolic pathways involved 3 ADs were identified (**Supplementary Tables S38–S40**). Specifically, “Arginine biosynthesis”, “Aminoacyl-tRNA biosynthesis”, “Arginine and proline metabolism”, “Alanine, aspartate and glutamate metabolism”, “Butanoate metabolism”, “Cysteine and methionine metabolism”, “D-Glutamine and D-glutamate metabolism”, “Glycine, serine and threonine metabolism”, “Nitrogen metabolism pathways”, “Valine, leucine and isoleucine biosynthesis” and “Valine, leucine and isoleucine degradation” were noticed to be involved in the pathogenetic process of RA. Meanwhile, SLE is observed to be associated with the “Aminoacyl-tRNA biosynthesis”, “alpha-Linolenic acid metabolism”, “D-Glutamine and D-glutamate metabolism”, “Glycerophospholipid metabolism”, “Linoleic acid metabolism”, “Nitrogen metabolism”, “Valine, leucine and isoleucine degradation” and “Valine, leucine and isoleucine biosynthesis” pathways. And T1D is associated with the “alpha-Linolenic acid metabolism”, “Arginine biosynthesis”, “Glycerophospholipid metabolism” and “Linoleic acid metabolism” pathways.

### 3.6 Differential plasma glutamate concentrations in normal controls and ADs patients

**Figure 9:**
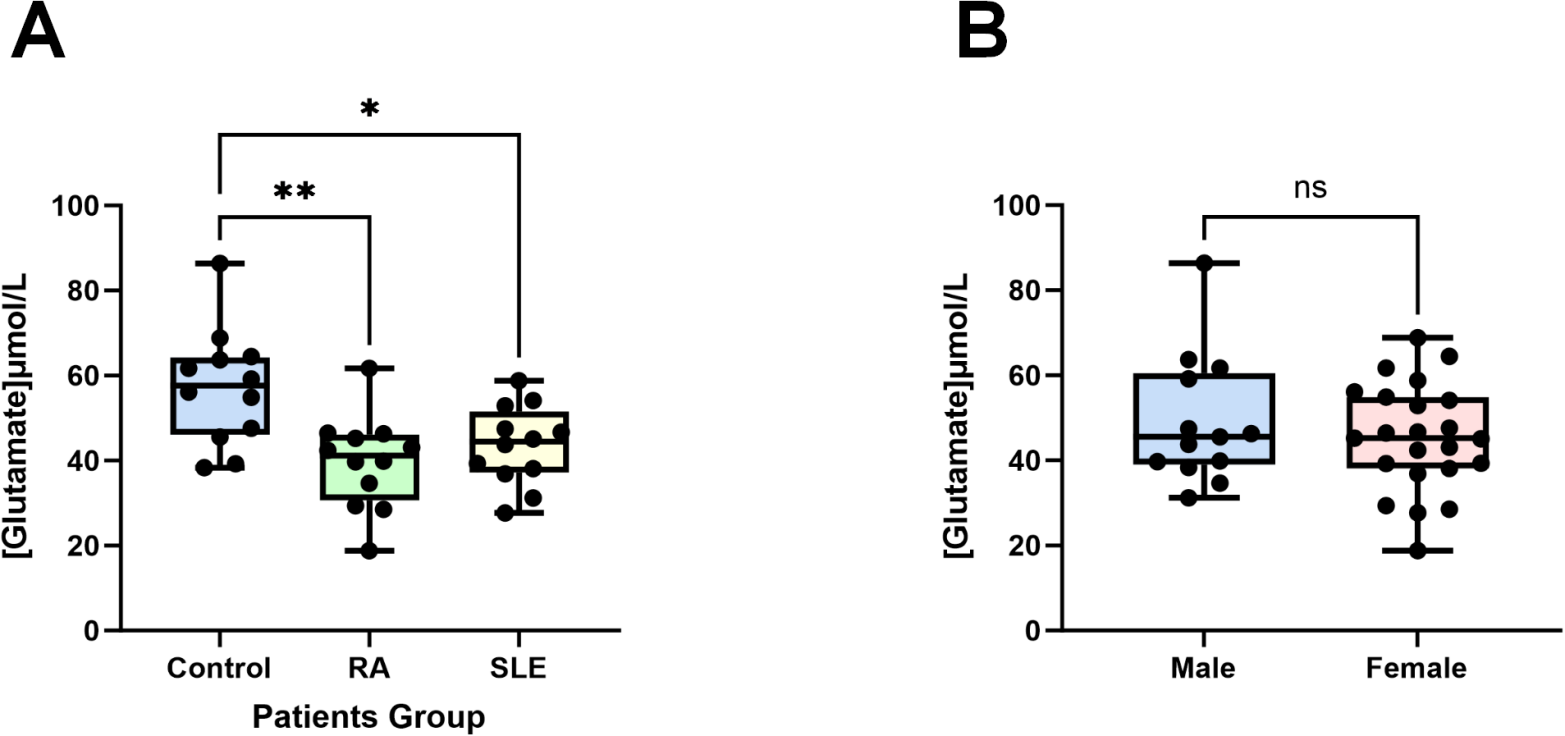
Glutamate concentrations in patients’ plasma. **(A)** Compared to the control group (healthy individuals, n = 12), the plasma glutamate concentrations in patients with RA (n = 12) and SLE (n = 12) were significantly reduced. **(B)** There are no differences in male (n = 13) and female (n = 23) plasma glutamate concentrations. Data are presented as the mean ± SD; ns: not significant, *P < 0.05, **P < 0.01.

To validate our findings, we experimentally confirmed the role of glutamate, identified as a protective factor in both SLE and RA from our previous results (**Figures 5**, **6**). In previous studies, glutamate has been identified as a pivotal excitatory neurotransmitter in the central nervous system, essential for cognitive function, memory, mood regulation, stress response, and immune modulation via its anti-inflammatory properties and involvement in glutathione synthesis (36–39). Using an ELISA kit, we found that the glutamate concentration in the control group (healthy individuals) was the highest at 57.18 ± 13.54 µM, significantly higher than those in the RA group (39.70 ± 10.95 µM, *P* < 0.01, n=12) and the SLE group (43.52 ± 9.321 µM, *P* < 0.05, n=12) (**Figure 9A**). Additionally, no significant differences in glutamate concentrations were observed between male and female plasma (**Figure 9B**). The consistency of these experimental results with our MR data corroborates the reliability and robustness of our findings.

## 4 Discussion

During this study, a bidirectional two-sample MR analysis was performed to assess the reciprocal causal correlation between plasma metabolites and 5 prevalent ADs (IBD, MS, T1D, SLE and RA). Furthermore, the results of pleiotropy at the gene level were excluded by sensitivity analysis, rendering the results of the study more robust and reliable. Through the Combination of evidence from both discovery and replication samples, we identified 4 metabolites that have causal relationships with IBD, 2 with MS, 13 with RA, and 4 with T1D. In the reverse MR study, we recognized causal relationships between SLE and 22 metabolites, IBD and 4 metabolites, RA and 22 metabolites, and T1D and 37 metabolites.

For the last few years, ADs have been increasingly acknowledged as a metabolism-related disease and changes in metabolites related to various ADs have been continuously discovered through metabolomics (12, 40–43). However, the existing studies have not been able to clarify the specific relationship between metabolites and ADs. Our research not only confirmed the presence of specific plasma metabolites causally related to ADs but also identified key metabolic pathways of ADs. The finding offered new perspectives into the prediction and diagnosis of ADs, along with providing potential targets for precision treatment.

In the results of the study, we first focused on the causality between lipid metabolites and ADs. Our research results have shown that there is a causal relationship between lipid-related metabolites and every type of ADs. In our study, sphingosine was found to be a protective factor for IBD. Sphingosine can generate S1P through a phosphorylation reaction, which is a bioactive lipid molecule (44). Several studies have shown that sphingosine-1-phosphate (S1P) has a regulatory effect in IBD, and selective S1P agonists have anti-inflammatory properties, which also indirectly validated our study (45, 46). These findings suggested novel targets for IBD therapy, and our study provided further support for this notion, highlighting the crucial role of sphingosine as a protective factor in the management of IBD progression.

In addition, our study indicated that 11 plasma metabolites of the lipid metabolism pathway participated in the pathogenesis of RA. In another study of inflammatory bowel disease serum metabolome, three metabolites overlapped with ours. Contrary to their study, we believed that 1-oleoyl-2-linoleoyl-GPE (18:1/18:2)* and 1-stearoyl-2-linoleoyl-GPE (18:0/18:2)* are protective factors for RA, but they confirmed that they are risk factors for Crohn’s disease. Interestingly, as revealed in our study, 1-stearoyl-2-arachidonoyl-GPC (18:0/20:4) was considered to be a risk factor for RA while they indicated it would be a protective factor for Crohn’s disease (47). Although the causality between these metabolites and different diseases is not completely consistent, it is jointly confirmed that these metabolites do have a causal connection with the occurrence and development of autoimmune diseases.

Our results also found a causal relationship between alterations in other metabolites involved in lipid metabolism and ADs, for example, for T1D, glycohyocholate is a risk factor. In multiple metabolomic studies related to asthma, it has also been observed that there are significant differences in levels of glycohyocholate between diseased children and the control group (48, 49). In a study on nonalcoholic fatty liver disease, after all confounding factors are excluded, the proportion of glycohyocholate remains significantly correlated with the presence of nonalcoholic fatty liver disease (50). All these pieces of evidence collectively indicated the causal link between abnormal levels of glycohyocholate and the occurrence of autoimmune-related diseases.

Undoubtedly, lipid metabolism imbalance plays a crucial role in the self-immune response, although the specific mechanisms by which lipid metabolism imbalance leads to inflammatory reactions and aberrant activation of immune system remain unclear (51–53). Based on these known evidences and our findings, these plasma metabolites may hold therapeutic targets for the treatment of ADs and promising research direction.

At the same time, not only in lipid metabolism, changes in other metabolites belonging to other pathways such as metabolites from cofactors and vitamin metabolism pathway are also causally related to ADs. According to our research, threonate is a protective factor for MS. As a chronic autoimmune disease, MS mainly impacts the central nervous system, which includes brain and spinal cord (54). In previous studies, threonate was often used as an important component of L-Threonic acid Magnesium salt, which was crucial in the treatment of orthopedic diseases and Alzheimer’s disease (55–57). Research findings point that threonine is a unique molecule that effectively regulates the structural and synaptic architecture and function of the central nervous system (58). According to the research, supplemental intake of threonate may help improve neurodegenerative conditions and cognitive function in MS patients. Correspondingly, threonate also has antioxidative and anti-inflammatory properties, which may potentially benefit the prevention and treatment of MS.

Furthermore, pathway analyses revealed diverse metabolic pathways related to autoimmune responses. Our results have shown that metabolic pathways involved in autoimmune diseases are often not singular, proving that an AD is often associated with multiple metabolisms. Furthermore, we have observed an overlap of metabolic pathways among various autoimmune diseases, indicating a tendency for the metabolic pathways involved in ADs to intersect. According to our research, “Glycerophospholipid metabolism”, “alpha-Linolenic acid metabolism” and “Linoleic acid metabolism” pathways were all involved in the pathogenetic process of RA, SLE and T1D. Interestingly, all of these metabolic pathways are interconnected with lipid metabolism. Lipid metabolites, as elucidated above, exert a pivotal influence in the etiology and progression of autoimmunity disorders. In a study based on multi-omics analysis, glycerophospholipid metabolism was revealed to play a key role in the occurrence and development of RA (59). Similarly, in another UPLC-MS/MS-based plasma lipidomics study, glycerophospholipid metabolism of lipid metabolites was demonstrated to be upregulated in SLE patients, further supporting our findings (60). Meanwhile, the previous study has reported the amelioration of ω-3 polyunsaturated fatty acids on T1D (61). Alpha-Linolenic acid, as a type of ω-3 polyunsaturated fatty acids, aligns with our findings, providing further evidence that this metabolic pathway plays a significant role in ADs. Equally, through another MR Study, the researchers demonstrated that linoleic acid exerts a protective function in the development of RA and SLE (62). Human body can consume these lipids from common food, so these metabolism researches provided dietary guidelines for the prevention of ADs. The opportunity for individuals to prevent ADs to some extent through the moderate intake of lipids in the daily dietary makes this discovery highly significant. Combined with previous perceptions, we speculated that it may be due to the occurrence of metabolic pathway disorders, especially lipid related metabolism disorders, leading to metabolic changes and immune dysfunction (63–66). Whereas, it also suggests that we can get new directions for prevention and treatment of ADs through the intervention of these metabolic pathways and crucial metabolites in the pathways.

At the level of genetic variation, identifying the causal effects of metabolites and metabolic pathways on ADs reveals potential targets for treatment or intervention. Subsequent validation in cellular and animal models, such as gene overexpression or knockdown experiments, along with metabolite intervention studies, can further confirm these causal relationships. Based on these findings, the development of drugs targeting specific metabolites or pathways can be pursued, potentially involving small molecule compounds or biologics to modulate the activity of these metabolites. On the other hand, understanding the causal influence of disease on metabolites enables the proactive prediction of individuals at risk of developing autoimmune conditions. Further development of risk scoring systems or the assessment of representative biomarkers can be employed to achieve this. According to the results of these risk predictions, dietary advice or pharmacological interventions can be provided to high-risk individuals to delay or prevent the onset of ADs. Of course, the efficacy and safety of these interventions need to be tested in human subjects through randomized controlled trials and other clinical study designs. After clinical application, long-term monitoring of high-risk individuals can be conducted to assess the onset and the long-term effects of intervention measures in real-time.

There were several strengths to this MR study. First, the most up-to-date and extensive databases of metabolites were selected. Meta-analysis of the two groups of data was conducted employing METAL software, resulting in 1,009 metabolites obtained through intersection (20). Meanwhile, the whole-genome data of 5 common ADs were acquired from 10 GWAS datasets, with each AD consisting of discovery and validation sets. This enabled us to conduct a comprehensive and systematic analysis of the metabolic profiles associated with the development of ADs. Second, our MR design investigated the causal relationship between plasma metabolites and ADs from the perspective of forward and reverse respectively, analyzing both the influence of metabolites on ADs and the impact of ADs on metabolites, which are mutually causal. This design facilitates the study more comprehensive, while the extensive sensitivity analysis ensures the reliability and robustness of our inferences. Ultimately, we conducted ELISA assays using plasma samples from the patients. The findings from these assays were in concordance with the results of the MR analysis, further substantiating the reliability of our analytical outcomes.

Our study still possessed certain limitations. Firstly, our study reported the causal relationship between ADs and metabolites, covering a relatively comprehensive metabolite spectrum, but the mechanism and function of many metabolites in diseases have not been fully elaborated, and require further investigation. Secondly, the data primarily originated from a European population, which limits the extrapolation of our results across ethnic groups. Thirdly, beyond the ELISA results, we did not explore gender disparities within our study. To address this gap, future research should incorporate a gender-based analysis and consider additional factors associated with gender, thereby enhancing our understanding of the mechanisms underlying ADs.

## 5 Conclusions

From the perspective of genetics, our systematic investigation revealed the causal relationship between plasma metabolites and diverse ADs, offering valuable insights into their etiology and underlying mechanisms. Furthermore, our findings provided potential inspiration for further accurate diagnosis and precision treatment strategies.

## Data Availability

The original contributions presented in the study are included in the article/**Supplementary Material**. Further inquiries can be directed to the corresponding authors.
