# Supplementary material for: Causal association between plasma metabolites and diverse autoimmune diseases: a two-sample bidirectional mendelian randomization study
Source: Front Immunol. 2024 Nov 7;15:1437688. doi: 10.3389/fimmu.2024.1437688 (PMC11578997; doi:10.3389/fimmu.2024.1437688)
Supplement: Supplementary file 1 [file DataSheet1.docx]

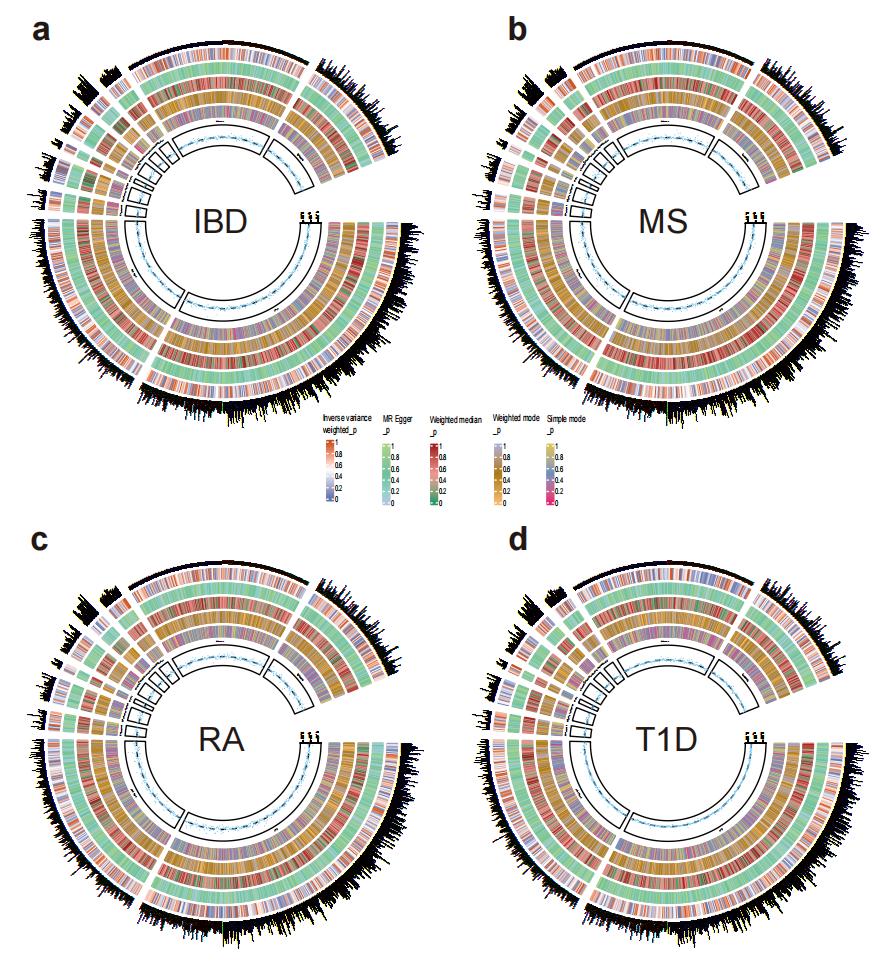


**Supplementary Figure 1.** Circle diagrams of the replication sample in the forward MR analysis. The complete results of the forward MR analysis, showing the causal effects of plasma metabolites on ADs. (a). IBD, (b). MS, (c). RA, (d). T1D. Five statistical methods, respectively, IVW, MR Egger, Weighted median, Weighted mode, and Simple mode, are represented by five circles from outer to inner.


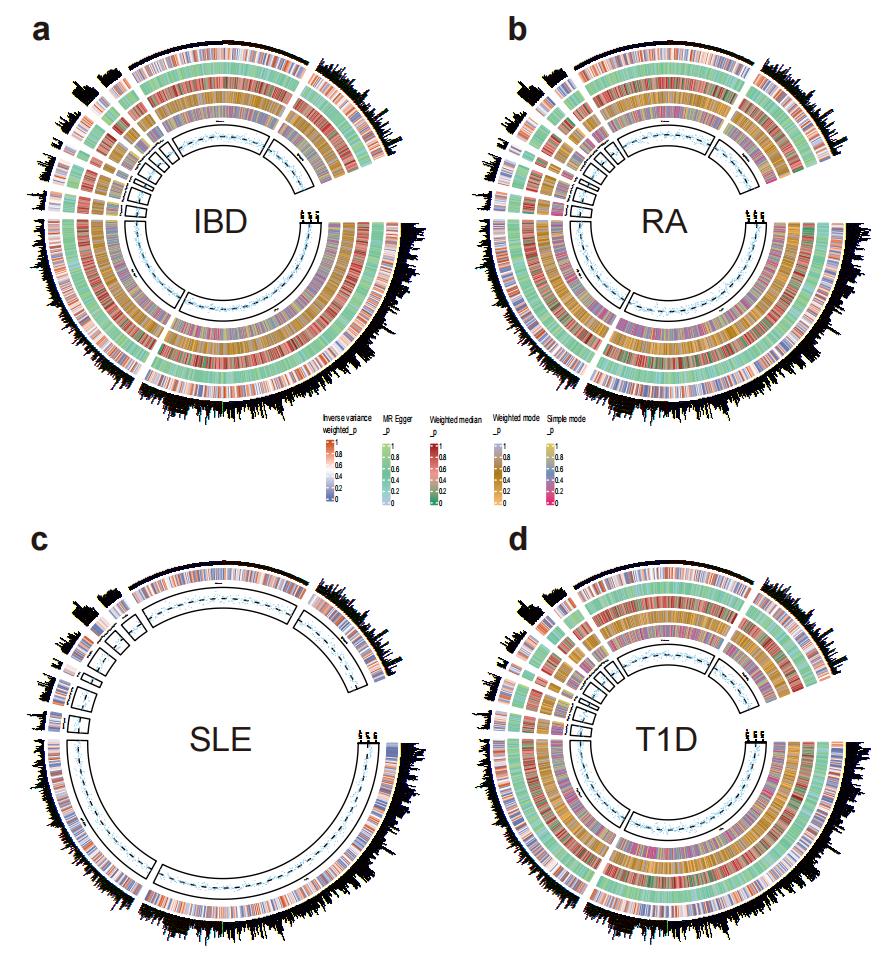


**Supplementary Figure 2.** Circle diagrams of the replication sample in the reverse MR analysis. The complete results of the reverse MR analysis, showing the causal effects of plasma metabolites on ADs. (a). IBD, (b). RA, (c). SLE, (d). T1D. Five statistical methods, respectively, IVW, MR Egger, Weighted median, Weighted mode, and Simple mode, are represented by five circles from outer to inner.

**
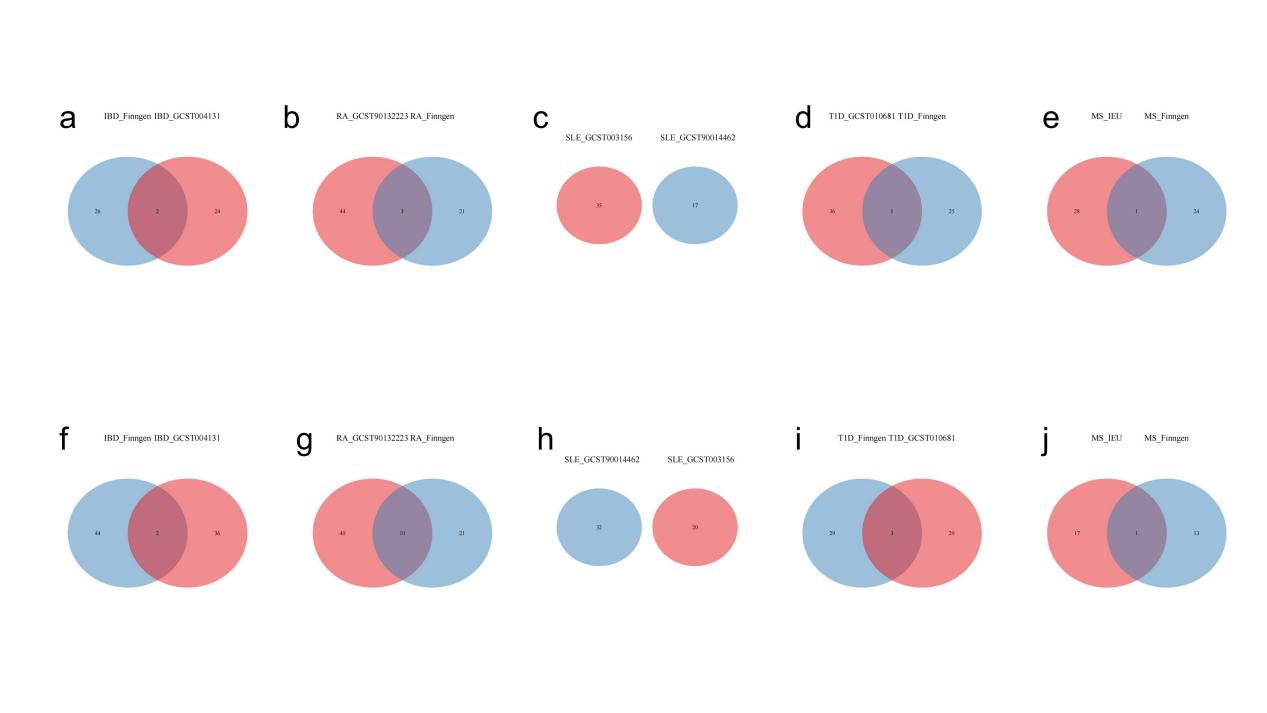
Supplementary Figure 3.** Intersection of discovery and replication sets in the forward MR analysis. (a-e). Metabolites were identified as risk factors simultaneously. (a). IBD, (b). RA, (c). SLE, (d). T1D, (e). MS. (f-j). Metabolites were identified as protective factors simultaneously. (f). IBD, (g). RA, (h). SLE, (i). T1D, (j). MS.


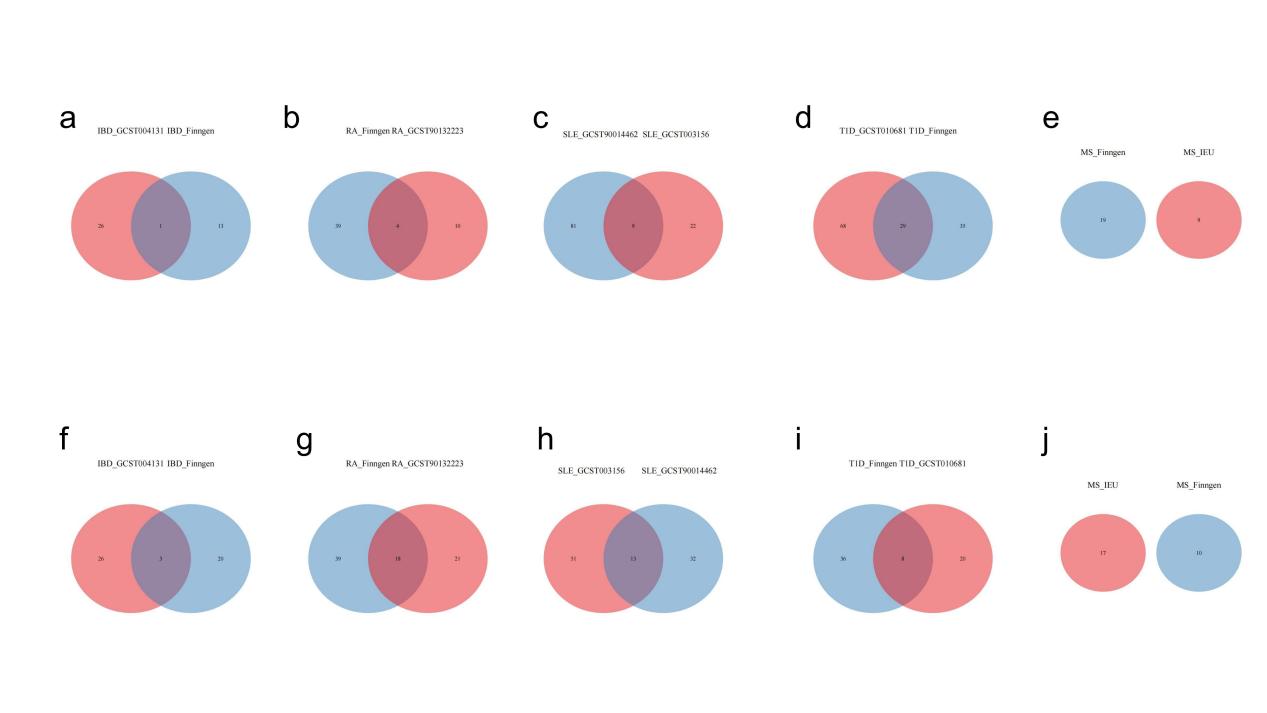


**Supplementary Figure 4.** Intersection of discovery and replication sets in the reverse MR analysis. (a-e). Metabolites were identified as risk factors simultaneously. (a). IBD, (b). RA, (c). SLE, (d). T1D, (e). MS. (f-j). Metabolites were identified as protective factors simultaneously. (f). IBD, (g). RA, (h). SLE, (i). T1D, (j). MS.
